# The E1a Adenoviral Gene Upregulates the Yamanaka Factors to Induce Partial Cellular Reprogramming

**DOI:** 10.3390/cells12091338

**Published:** 2023-05-07

**Authors:** Gracia Mendoza, Rebeca González-Pastor, Juan Miguel Sánchez, Altamira Arce-Cerezo, Miguel Quintanilla, Gema Moreno-Bueno, Anna Pujol, Carolina Belmar-López, Alba de Martino, Efrén Riu, Tristan A. Rodriguez, Pilar Martin-Duque

**Affiliations:** 1 Instituto Aragonés de Ciencias de la Salud (IACS), 50009 Zaragoza, Spain; 2 Instituto de Investigación Sanitaria Aragón (IIS Aragón), 50009 Zaragoza, Spain; 3 Centro de Investigación Biomédica (CENBIO), Facultad de Ciencias de la Salud Eugenio Espejo, Universidad UTE, Quito 170527, Ecuador; 4 National Heart and Lung Institute, Imperial College London, London W12 ONN, UK; 5 Centro de Biotecnología Animal y de Terapia Génica (CBATEG), Universidad Autónoma de Barcelona, 08193 Bellaterra, Spain; 6 Departamento de Bioquímica, Instituto de Investigaciones Biomédicas ‘Alberto Sols’, Universidad Autónoma de Madrid (UAM), (UAM-CSIC), 28029 Madrid, Spain; 7 Fundación MD Anderson Internacional, 28033 Madrid, Spain; 8 Centro de Investigación Biomédica en Red, Instituto de Salud Carlos III, Red de Cáncer (CIBERONC) and Red de Nanomedicina y Nanomateriales (CIBER-BBN), 28029 Madrid, Spain; 9 OncoGenomics Lab, Universidad Privada San Juan Bautista, Lima 15038, Peru; 10 Fundación Araid, 50018 Zaragoza, Spain; 11 Departamento de Cirugía, Facultad de Medicina, Universidad de Zaragoza, 50009 Zaragoza, Spain

**Keywords:** reprogramming, induced pluripotent stem cells, embryonic stem cells, Yamanaka factors, adenovirus, E1a gene

## Abstract

The induction of pluripotency by enforced expression of different sets of genes in somatic cells has been achieved with reprogramming technologies first described by Yamanaka’s group. Methodologies for generating induced pluripotent stem cells are as varied as the combinations of genes used. It has previously been reported that the adenoviral E1a gene can induce the expression of two of the Yamanaka factors (c-Myc and Oct-4) and epigenetic changes. Here, we demonstrate that the E1a-12S over-expression is sufficient to induce pluripotent-like characteristics closely to epiblast stem cells in mouse embryonic fibroblasts through the activation of the pluripotency gene regulatory network. These findings provide not only empirical evidence that the expression of one single factor is sufficient for partial reprogramming but also a potential mechanistic explanation for how viral infection could lead to neoplasia if they are surrounded by the appropriate environment or the right medium, as happens with the tumorogenic niche.

## 1. Introduction

Cellular reprogramming of somatic cells has been one of the fundamental questions in stem cell biology. A major breakthrough in this field was achieved when it was demonstrated that embryonic and adult somatic cells could be reprogrammed to the pluripotent state just by overexpression of four defined genes, c-Myc, Klf4, Oct4, and Sox2 [1,2]. The combination of these four factors has been subsequently termed the Yamanaka factors, and the resulting reprogrammed cells are defined as induced pluripotent stem (iPS) cells. This ability to reprogram somatic cells has created enormous expectations, given the potential clinical applications of these iPS cells. For example, to facilitate the generation of patient-specific stem cells that would overcome the ethical problems posed by human embryonic stem cells (ESCs) and reduce the risk of immune rejection [3]. For these reasons, stem cell researchers have invested significant efforts in improving both the efficiency and the technical limitations that accompany the reprogramming process. As part of this effort, a number of studies have reported various mechanisms for enhancing the efficiency and rate of reprogramming, for example, by using small molecules [4,5,6]. Another major effort has been to identify cell types where it is possible to reduce the number of Yamanaka factors used for reprogramming. In this way, some groups described that Oct4 expression might be sufficient to initiate and maintain the reprogramming process in early embryonic cell types or neural stem cells [7,8], while recently, Myc has been shown not to be required for the maintenance of pluripotency [9].

The adenovirus 5 early region 1 (E1) gene is involved in a large number of gene and growth regulatory activities, such as the stimulation of cell growth and the inhibition of differentiation (by deregulating the normal transcription pathways of the host cell) in order to obtain a productive infection [10]. In this E1 region, E1a is the first viral gene expressed in cells upon infection and encodes two major proteins of 289 (E1A-13S) and 243 (E1A-12S) residues that arise from differential splicing of the same transcript and differ only by the presence of a residue of 46 amino acids [11]. In addition, E1A interacts with many cellular proteins, including retinoblastoma protein (pRb), TBP, CBP/p300, p400, YY1, and CDK8, amongst others. All of these have individual contributions to the E1A function, although the interaction of E1A with chromatin remodeling proteins, such as CBP/p300 and p400, have also been suggested to be important for E1a function [12,13].

A number of studies have indicated a nexus between E1a and two of the Yamanaka factors: Oct4 and c-Myc [12,13,14,15]. The induction of c-Myc mediated by E1a is related to the binding to pRb, p300, and p400 host proteins [12,13]. Moreover, p300-binding has been shown to be essential for the transactivation of a third Yamanaka’s factor, such as Sox-2, with an indirect involvement of the E1a gene [16], and it has been reported as a key factor in the induction of S-phase and the loss of growth control [13,17]. More importantly, Oct4 transactivation is strongly stimulated by E1a in differentiated cells through the interaction with TATA box factors [14]. The ability of E1a to activate Oct4 and c-Myc in certain contexts, together with the E1a effects in histone modification patterns [18,19], led us to hypothesize that E1a overexpression alone may be sufficient to initiate the reprogramming process. Here, we demonstrate that mouse embryonic fibroblasts (MEF) can be induced into iPS-like cells, closely resembling epiblast stem cells (EpiSCs), by the presence of a single adenoviral gene, E1a-12S, although this process was maintained in combination with MEK and GSK3β small molecule inhibitors.

## 2. Materials and Methods

### 2.1. Cell Culture

Murine Embryonic Fibroblasts (MEF) were obtained from HP165 mice, which have a genetic background derived from 129 and MF1 (outbred) mice crossing [20]. Wild-type MEF cells were provided by Dr. Tristan A Rodríguez (Imperial College London, London, UK), while MEF Oct4-GPF cell lines were kindly provided by Dr. Jennifer Nichols (Wellcome Trust Centre for Stem Cell Research, University of Cambridge, Cambridge, UK). All MEFs, 293T, and 293 cell lines were cultured in Dulbecco’s Modified Eagle’s Medium (DMEM) containing 10% heat-inactivated fetal bovine serum (FBS) and 50 U penicillin/streptomycin. The media were changed twice a week. Reprogrammed MEFs were cultured under standard mouse embryonic stem cells (mES) conditions: Glasgow’s modified Eagle’s medium (GMEM) containing 10% heat-inactivated FBS, 50 U penicillin/streptomycin, 1% sodium pyruvate, 1% non-essential amino acids, 0.1% β-mercaptoethanol, and 100 U of leukemia inhibitory factor (LIF) (Invitrogen, Waltham, MA, USA). mES cells were collected from E14 embryos and characterized by Dr. Tristán Rodríguez (Imperial College London, UK). mES and induced pluripotent stem (iPS)-like cells were seeded on tissue culture plates coated with 0.1% gelatin and cultured with mES standard medium supplemented with MEK (1 µM; Merck; Rahway, NJ, USA) and GSK3β (3 µM; Stemgent, Boston, NJ, USA) inhibitors [6]. Media were replaced every other day. All the experiments were performed when cells were cultured in this conditioned medium for 25–45 days. All the media and FBS used were purchased from Gibco (Waltham, MA, USA), while the other cell culture supplements were obtained from Lonza (Durham, NC, USA). All the cell lines used in this study were cultured at 37 °C and 5% CO_2_.

### 2.2. E1a Retroviral Reprogramming of MEF Oct4-GFP

MEF Oct4-GFP were plated 48 h prior to infection. A total of 50,000 cells per well were seeded on a 6-well plate, previously coated with 0.1% gelatin. Polybrene (4 mg/mL) was added to the viral supernatant in order to increase the transduction efficiency. The medium was removed, and cells were incubated with 1 mL of retrovirus carrying the E1a-12S gene between 4–16 h. To prepare the retrovirus, the pLPC-E1a-12S [21] or the pLPC-GFP plasmids (kindly donated by Dr. Ramón y Cajal) were used. Maps and further explanation regarding the E1a construct could be found at the addgene site (https://www.addgene.org/18740/sequences/, accessed on 6 May 2023) or in supplemental data. Plates were centrifuged at 700× *g* for 45 min and 32 °C. The infectious medium was removed and replaced with fresh medium. After 72 h, 50,000 reprogrammed cells were plated on feeder cells seeded on gelatinized 10 cm dishes and cultured with mES standard medium. To test GFP expression and, thus, the efficiency of reprogramming, iPS-like (GFP expressing or not) clones were visualized 14 days after transfection in a fluorescence microscopy IX81 Olympus equipped with an XC50 Olympus camera, and images were processed using Cell^D 5.1 software (Olympus, Shinjuku, Japan). Then, single clones were transferred into gelatinized 96-well flat-bottomed tissue culture plates containing 50 µL of fresh medium. Previously, clones were picked with a 10 µL micropipette from the 10 cm dishes to 96-well round-bottomed plates containing 25 µL of Trypsin/EDTA. Clones were disaggregated with trypsin, then transferred into the gelatinized 96-well flat-bottomed plates. mES standard medium was changed every day. In order to expand the clones, four gelatin-coated 48 well-plates were used per each 96 well-plate. Cells were washed with PBS, trypsinized with 50 µL of Trypsin/EDTA and 200 µL of fresh medium was added per well. Clones were disaggregated by pipetting, and 100 µL of cell suspension was transferred into a well of a 48 well-plate in duplicate. The medium was changed every day.

### 2.3. Alkaline Phosphatase Activity and E1a Expression

Alkaline phosphatase staining was performed both in iPS-like cell cultures and in embryoid bodies with the alkaline-phosphatase detection kit (Sigma, Waltham, MA, USA) according to the manufacturer’s protocol. Briefly, cells were fixed with the citrate-acetone-formaldehyde solution for 30 s at room temperature. Then, cells were washed and stained with the alkaline staining solution for 15 min at room temperature and, after washing, with hematoxylin for 2 min. The samples were evaluated in an IX81 Olympus equipped with an XC50 Olympus camera, and images were processed using Cell^D 5.1 software (Olympus, Shinjuku, Japan).

In order to evaluate whether the E1a gene was still activated in iPS-like cells, and could disrupt differentiation and embryogenesis processes, nested PCR (nPCR) and sequencing studies were performed. The detection of the E1a gene was analyzed by nPCR, as other authors described, in order to amplify the low presence of the E1a adenoviral gene in infected cells [22,23,24]. First, genomic DNA from 293 cells (positive control for E1a gene) and iPS-like cells was isolated with the Nucleospin Tissue extraction kit (Macherey-Nagel, Düren, Germany) following the manufacturer’s protocol. The PCR amplifications were performed in a final volume of 10 µL consisting of 1X PCR buffer (Bioline, London, UK), 1.5 mM MgCl2 (Bioline, London, UK), 0.2 mM deoxynucleoside triphosphate mix (Invitrogen, Waltham, MA, USA), 0.3 U of BIOTAQ DNA polymerase (Bioline, London, UK), 200 nM each primer (Sigma, Waltham, MA, USA; sequences designed with Oligo 7 Software (Molecular Biology Insights Inc., Colorado Springs, CO, USA) are shown in Supplementary Table S1), and 100 ng of template DNA, in a 2720 Thermal Cycler (Applied Biosystems, Waltham, MA, USA) for a total of 35 cycles. After an initial denaturation step for 2 min at 94 °C, each cycle consisted of denaturation for 20 s at 94 °C, annealing for 30 s at 61 °C, and primer extension for 30 s at 72 °C. Then, primary PCR products were used in a second amplification. nPCR products were visualized on a 2% agarose gel stained with ethidium bromide in a G:Box UV Transilluminator (Syngene, Bangalore, India), and images were displayed by the Gen5 10.9 Data Analysis Software (Biotek, Winooski, VT, USA). The primary PCR product was 383 bp, while the second product was 103 bp. Products of primary PCR were sequenced with a 3500xL Genetic Analyzer (Applied Biosystems, Waltham, MA, USA), and sequences were aligned by BioEdit v7.1.3 Software (Ibis Biosciences, Carlsbad, CA, USA) and compared to the E1a 12S region sequence from adenovirus 5 in GenBank.

### 2.4. Immunocytochemistry

mES and iPS-like cells were seeded on gelatin-coated glass coverslips in 24-well plates at 50,000 cells/well. After 24 h, cells were fixed in 4% paraformaldehyde for 20 min at room temperature and washed thrice with PBS. In the last washing, 50 µL ammonium chloride (Panreac, Chicago, IL, USA) 1 M was added per well in order to eliminate the remaining paraformaldehyde remaining residues. Then, cells were permeabilized with 100 µL/well PBS-0.5% Triton X100 (Biorad, Hercules, CA, USA) and incubated for 10 min at room temperature. Cells were rinsed and incubated in primary antibody containing 10% FBS (Gibco, Waltham, MA, USA) in PGBA (0.1% gelatin (Sigma, Waltham, MA, USA), 1% bovine serum albumin (Sigma), and 0.05% sodium azide (Sigma, Waltham, MA, USA) in PBS) at 4 °C overnight. Immunostaining was performed with the Mouse Embryonic Stem Cell Marker Panel Kit (Abcam, Cambridge, UK), which contains polyclonal rabbit anti-Oct4 (1:200), anti-Nanog (1:25), anti-Sox2 (1:50), anti-Lin 28 (1:1000), and mouse monoclonal anti-SSEA1 (1:10). Then, cells were rinsed and incubated in secondary antibody (donkey anti-rabbit or donkey anti-mouse Alexa488 (1:1000; Invitrogen, Waltham, MA, USA)) containing PGBA at room temperature for 20 min. After washing, coverslips were mounted on glass slides in DAPI-Mowiol mounting medium and subsequently sealed with nail varnish. Confocal images were collected using a Fluoview FV1000 Confocal Microscope (Olympus, Shinjuku, Japan), then processed with FV1000 Software and Adobe Photoshop SC 5.01 (Adobe Systems, San Jose, CA, USA).

### 2.5. Embryoid Bodies Generation

mES and iPS-like cells were trypsinized and resuspended in their conditioned medium at a density of 40,000 cells/mL. Embryoid body formation was induced by seeding 50 drops of the cell suspension (1000 cells per drop) on the lid of a cell culture dish 100 mm in diameter and adding PBS to the dish. The lid was turned, and the dish was incubated at 37 °C and 5% CO_2_. After 48 h, cells were collected with conditioned medium and transferred to a Petri dish to allow embryoid bodies growth in suspension for 5–7 days at 37 °C and 5% CO_2_. Then, the embryoid bodies were transferred to 0.1% gelatin-coated dishes, where they adhered for 2–3 days. The medium was changed every 3–4 days. The samples were visualized in an IX81 Olympus equipped with an XC50 Olympus camera, and images were processed using Cell^D 5.1 software (Olympus, Shinjuku, Japan).

### 2.6. RNA Isolation and qPCR Analysis

Total RNA was isolated from mES, iPS-like cells, MEF, and embryoid bodies with the NucleoSpin RNA II kit (Macherey-Nagel, Düren, Germany) according to the manufacturer’s protocol. Complementary DNA (cDNA) was obtained from 250 ng of total RNA with the iScript Reverse Transcription Supermix (Bio-Rad, Hercules, CA, USA) in a 2720 Thermal Cycler (Applied Biosystems, Waltham, MA, USA). Quantitative PCR (RT-qPCR) reactions of cDNA obtained from mES, iPS-like cells, and MEF were performed using 2 µL of a 2.5 ng of cDNA in a final reaction volume of 10 µL with the TaqMan Universal Master Mix II (no UNG) and different TaqMan probes in Applied Biosystems 7900HT Fast-Real Time PCR System (Applied Biosystems, Waltham, MA, USA). Pre-incubation step consisted of 1 cycle of 10 min at 95 °C. The amplification step comprised 40 cycles of 15 s at 95 °C and 1 min at 60 °C. Then, the plate was read. cDNA samples of embryoid bodies were analyzed using an OpticonIITM DNA engine and Opticon Monitor 3.1 software (MJ Research Inc., Deltona, FL, USA). PCR reactions included SYBR Green PCR Mastermix (Qiagen, Hilden, Germany), 300 nM primers, and 2 μL of diluted cDNA template in a 30 μL reaction volume. PCR conditions were as follows: 95 °C for 15 min, then 40 cycles of 94 °C for 15 s, 60 °C for 30 s, 72 °C for 30 s, followed by plate-read. Melting curves were calculated to check the specific amplification of the sequence of interest. The relative gene expression was determined by using the 2^−ΔΔCT^ comparative method. Expression values were normalized with 18S gene values in the pluripotency genes expression assay and in the validation of microarray analyses, while β-actin was used as a housekeeping gene in the differentiation experiments. Each measurement was performed in triplicate. Data were processed with the SDS2.2.2 Software (Life Technologies, Carlsbad, CA, USA). TaqMan probes (Life Technologies, Carlsbad, CA, USA) and primer sequences (Sigma, Waltham, MA, USA) are listed in the Supplementary information, respectively.

### 2.7. Gene Expression Profiling

MEF, mES, and iPS-like cells were harvested, RNA obtained, and cDNA transcribed as described above. Samples were hybridized onto the Whole Mouse Genome Oligo Microarray (Agilent Technologies, Santa Clara, CA, USA) following the manufacturer’s protocol. Briefly, samples were Cy-3 labelled (Quick-AMP Labelling Kit, Agilent Technologies, Santa Clara, CA, USA) and fragmented into pieces ranging from 35 to 200 bases. Fragmented cRNA samples (1.65 μg) were hybridized for 17 h at 65 °C on chips. Then, GeneChips were washed, dried, and scanned in an Axon 5000 XL Scanner (GSI Lumonics, Bedford, MA, USA), and data were processed using Feature Extraction Software 10.0 (Agilent technologies, Santa Clara, CA, USA). Microarray raw data tables have been deposited in the Gene Expression Omnibus. For statistical analysis, we selected genes whose expression differed by a factor of at least two with respect to control cells. A hierarchical clustering method was applied to group the genes and samples on the basis of the similarities in expression, and the unsupervised analyses were visualized using the Self-Organizing Tree Algorithm (SOTA) and TreeView software, assuming Euclidean distances between genes (http://bioinfo.cnio.es/cgi-bin/tools/clustering/sotarray accessed on 3 June 2016).

### 2.8. GeneChIP Assay

Chromatin immunoprecipitation (ChIP) experiments were performed on three biological replicates of MEF, mES, and iPS-like cell samples, using the HighCell# ChIP kit (Diagenode, Liege, Belgium) in accordance with the manufacturer’s instructions. Briefly, cultured cells were fixed with 1% formaldehyde for 10 min at room temperature, followed by quenching with 125 mM glycine for 5 min. This was followed by three washes with PBS and the addition of cell and nucleus lysis buffers to the cell extracts. The cells were then sonicated using a Sonics Vibra-Cell VC130 sonicator (14 × 30 s on ice with 1 min waiting on ice between pulses; Diagenode, Liege, Belgium) to shear the cross-linked chromatin into an average DNA fragment size of 200–600 bp. Immunoprecipitation was performed overnight with agitation at 4 °C with 50 µg of chromatin using 5 µg of antibodies against H3K27me3 (trimethylated Lys27 of histone 3, #07-449, Millipore, Burlington, VT, USA), H3K4me3 (trimethylated Lys4 of histone 3, #003-050, Diagenode, Liege, Belgium), and Acetylated H3 (acetylated histone H3, #06-599, Millipore, Burlington, VT, USA) or unspecific rabbit IgG (#12-370, Millipore, Burlington, VT, USA) as a control. After overnight incubation, the DNA-protein-antibody complex was eluted, and the cross-links were reversed by adding elution buffer containing proteinase K followed by incubation of the samples for 4 h at 65 °C. DNA was extracted by the phenol-chloroform-isoamyl alcohol, ethanol-precipitation, and resuspended in water. qPCR analysis was performed by RT-qPCR with the LC480 system (Roche, Basilea, Switzerland) with 2 µL DNA and SybrGreen master mix and the primers listed in Supplementary Table S4. The amplification program of qPCR consisted of denaturation at 95 °C for 5 min, followed by 45 cycles at 95 °C for 10 s denaturation, 60 °C for 10 s annealing, and 72 °C for 10 s extension. The relative proportions of immunoprecipitated promoter fragments were determined based on the threshold cycle (Ct) value for each PCR reaction. RT-PCR data analyses were obtained using the comparative Ct method using the formula: %(ChIP/Input) = 2^[(Ctinput − 6.644) − CtChIP]*100 (where 6.644 is the log2 of 100 and corrects for the use of 1% input material in the qPCR determining the Ctinput). Data from each IP were quantitated in duplicate for at least three separate experiments and calculated and expressed as a percentage of their corresponding inputs (1/10 dilution) of chromatin before immunoprecipitation.

### 2.9. In Vivo Studies

#### 2.9.1. Teratoma Formation

All procedures were performed in accordance with the Spanish Policy for Animal Protection RD1201/05. For teratoma induction, six- to eight-week-old female BALB/c nu/nu mice (Envigo RMS Spain, Barcelona, Spain) received subcutaneous (s.c) injections of 2 × 10^5^ mES or iPS-like cells in 200 µL of 1:10 Matrigel solution in PBS (Basement Membrane Matrix, High Concentration (HC), BD Bioscience, Franklin Lakes, NJ, USA) (*n* = 4/group). As described above, iPS-like cells were generated from MEFs obtained from HP165 mice [20]. When these tumors reached a volume of 100 mm^3^, teratoma tissues were harvested, fixed in 4% paraformaldehyde overnight and embedded in paraffin. The differentiation into cells of the three germ layers was evaluated by hematoxylin and eosin staining and by the histopathological stainings described below. The samples were evaluated in an IX81 Olympus equipped with an XC50 Olympus camera, and images were processed using Cell^D software (Olympus, Shinjuku, Japan).

#### 2.9.2. Chimera Production

First, the karyotype of the cells was studied. On the first day, two million cells were seeded without feeders for the karyotype at 24 h and for the karyotype at 48 h. Before the karyotyping of the cells medium was changed 2 h after the medium change, 8 μl of demecolcine stock solution in 4 mL of medium (at final concentration 0.02 μg/mL) was added and incubated for a maximum of 3 h at 37 °C and 5% CO_2_. Then, cells were trypsinized and centrifuged at 1200–1400 rpm before the supernatant was aspirated. Afterwards, KCl 0.56% was added twice over the pellet mixing before incubating for 20 min at rt. Cells we fixed and washed them before assembling the slides. Finally, cells were stained with Giemsa dye. For the chromosome counting, the Ikaros program was used, counting up to 50 metaphases.

Chimera production was carried out by the injection of reprogrammed cells to blastocysts and 8-cell embryos obtained from superovulated female mice (C57BL/6JOlaHsd) by administration of pregnant mare serum gonadotropin (PMSG), followed by the injection of human chorion gonadotropin (hCG). All blastocysts and 8-cell embryos were collected after the detection of vaginal plugs at 3.5 dpc and 2.5 dpc, respectively. Microinjected blastocysts were transferred to the uterus of pseudo-pregnant females of 2.5 dpc and 8-cell embryos to the oviduct of pseudo-pregnant females of 0.5 dpc.

### 2.10. Histological Studies

The immunohistochemistry reactions were performed in an automated immunostaining platform (Discovery ULTRA, Ventana, Roche Roche, Basilea, Switzerland ). Antigen retrieval was first performed with the appropriate pH buffer (CC1 buffer), and endogenous peroxidase was blocked (peroxide hydrogen at 3%). Then, slides were incubated with the appropriate primary monoclonal antibodies: Actin (mouse HHF35, Dako, Santa Clara, CA, USA), NeuN (mouse A60, Millipore MAB 377), Vimentin (Rabbit D21H3, Cell signaling technology 5741), Cytokeratin, or AE1/AE3 (pankeratin) (Mouse (AE1/AE3) Thermo scientific MS-343-P0). After the primary antibody, slides were incubated with the corresponding secondary antibodies when needed (anti-mouse Abcam), and visualization systems (OmniMap anti-Rabbit, Ventana, Roche, Basilea, Switzerland) conjugated with horseradish peroxidase.

An immunohistochemical reaction was developed using 3, 30-diaminobenzidine tetrahydrochloride (DAB) (ChromoMap DAB, Ventana, Roche, Basilea, Switzerland), and nuclei were counterstained with Carazzi’s hematoxylin. Finally, the slides were dehydrated, cleared, and mounted with a permanent mounting medium for microscopic evaluation. Positive control sections known to be primary antibody positive were included for each staining run.

Whole slides were acquired with a slide scanner (AxioScan Z1, Zeiss, Jena, Germany), and images were captured with the Zen Blue Software (V3.1 Zeiss).

### 2.11. Statistical Analyses

Results are reported as mean ± SEM. Statistical evaluation of data was carried out using the SPSS Statistics 17.0 software package (IBM, Armonk, NY, USA). The normal distribution of the variables was analyzed by the Kolmogorov–Smirnov test followed by the *t*-test or analyses of variance (ANOVA). *p* < 0.05 was considered to be statistically significant.

## 3. Results

### 3.1. Generation and Characterization of iPS-like Cells from MEFs by E1a-12S Overexpression

**Figure 1:**
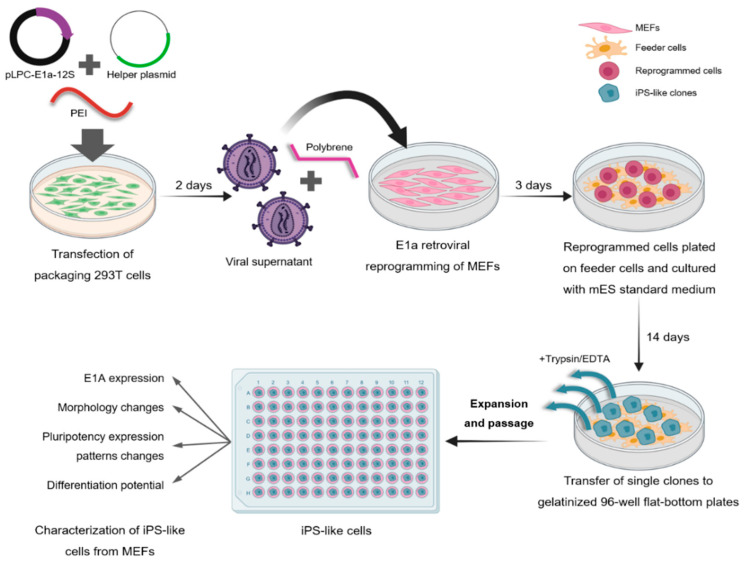
Schematic overview of E1a retroviral reprogramming of MEF Oct4-GFP. MEFs: mouse embryonic fibroblasts.

To establish E1a-12S as a potential reprogramming factor, first, we tested whether we could generate iPS cells from MEFs-Oct4GFP by simple overexpression of this viral gene. A schematic overview of the methodology followed is shown in Figure 1. As control of the morphology changes and the colonies formation, the same plasmid encoding GFP has been used for the control retroviral infections, although no colonies appeared on the previously mentioned controls (Figure S1). 

**Figure 2:**
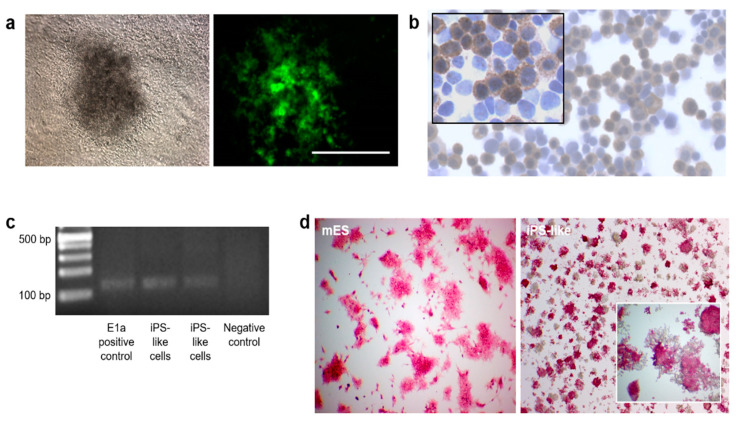
Characterization of iPS-like cells from MEFs by E1a-12S overexpression. (**a**) Bright-field (**left**) and fluorescence (**right**) images of the E1a-transduced cells after 14 days of infection. Scale bar = 300 μm. (**b**) Immunocytochemistry of E1A protein (200× and inset 400×). (**c**) PCR genotyping (**right**) of E1a gene in iPS-like cells after reprogramming. A total of 293 cells were used as E1a positive control. (**d**) Immunostaining for alkaline phosphatase on embryoid bodies (**left**) and iPS-like emerging colonies (**right**) (4× and inset 10×).

At 14 days after infection, the transduced cells began to cluster into colonies, displayed a typical mES cell-like morphology and showed GFP expression (Figure 2a and Figure S1). The colonies were picked to establish cell cultures. Recent studies have shown the potential of E1a together with Oct4 and Klf4 to generate iPS colonies, pointing to the possibility of replacing exogenous Sox2 to achieve reprogramming, though not E1a alone or E1a together with Oct4 [25]. Additionally, small molecules, such as MEK and GSK3 inhibitors, were used to increase reprogramming efficiency and quality by preventing ESCs from differentiating, restoring cell growth, and enhancing cell viability [6,26]. Together, these observations suggested that E1a-12S was inducing some kind of cellular reprogramming, and we named these cells iPS-like. However, in order to confirm that iPS-like cells were indeed transformed by E1a-12S, immunocytochemistry, nested PCR (nPCR), and sequencing of nPCR amplification products were performed (Table S1), showing that reprogrammed cells carried the E1a gene (Figure 2b,c). nPCR was developed in order to increase the specificity and sensitivity of PCR, as previously described for adenoviral genes [27,28,29].

After 25 days of culture, iPS-like cells grew stably in the 2i culture medium and clearly developed colonies. Then, similarities between iPS-like cells and ES cells were studied. Once in culture, the morphology of reprogrammed cells resembled more that exerted by mES than that of MEFs, as shown in Figure S2a. Moreover, as E1a was described to induce changes in the cell cycle [13,17], analysis of the growth abilities of these cells indicated that for the first 72 h after plating, their proliferation pattern was similar to mES cells. However, between 72 and 120 h, the iPS-like cells nearly doubled the growth rate of mES cells, suggesting that they maintain other characteristics to those found in the naïve pluripotent state (Figure S2b). In spite of this difference, we observed that the cell cycle profile of the iPS-like cells bared a much closer resemblance to that of mES than to the parental MEF cell line (Figure S2c).

Other similarities between mES and reprogrammed cells were also revealed by alkaline phosphatase staining, which was strongly displayed in iPS-like emerging colonies (Figure 2d). Furthermore, these cells showed their capacity to generate embryoid bodies (EBs) in suspension and in adhesion and growth in gelatin-coated cell culture dishes (Figure S3), which were capable of staining for alkaline phosphatase as well (Figure 2d). MEFs were used as negative controls, and, as expected, EBs were not obtained.

### 3.2. E1a Derived iPS-like Cells Are Similar to mES Cells at the Molecular Level

**Figure 3:**
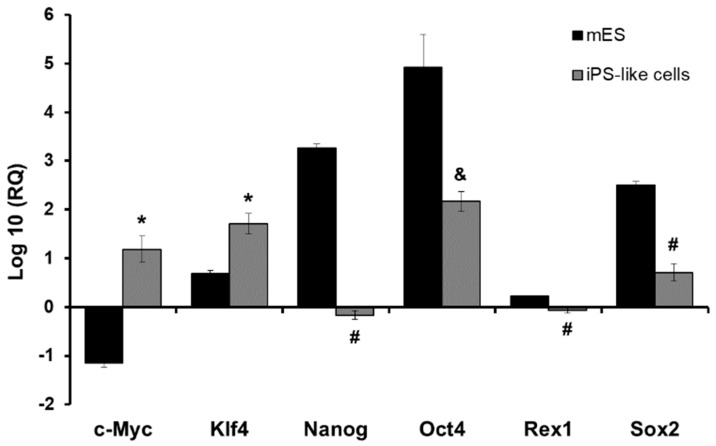
Molecular characterization of iPS-like cells. RT-qPCR of pluripotency genes was performed in mES, E1a-reprogrammed, and MEF cells. Expression values were normalized to the housekeeping 18S gene and expressed related to the control sample (MEF) expression (log(RQ) = 0, where RQ = relative quantification). Error bars represent mean ± SEM (*n* = 3). Significant differences between markers from each sample are denoted as * iPS-mES—, # iPS-mES, and & iPS-MEF.

In order to establish the degree to which the iPS-like cells were reprogrammed, core pluripotency genes expression was analyzed by real-time quantitative PCR (RT-qPCR) revealing robust expression of Oct4, Sox2, Klf4, and c-Myc (Table S2, Figure 3). In ESCs, a specific set of transcription factors (such as Nanog, Klf4, Oct4, and Sox2) act as part of a pluripotent gene regulatory network (GRN) to maintain pluripotency [30,31,32,33], while c-Myc is able to monitor ESCs proliferation and biosynthesis, but no pluripotency maintenance and identity, which can be maintained by the in vitro 2i culture [9]. In contrast, we found that iPS-like cells showed reduced expression of markers of the naïve state of pluripotency, such as Nanog and Rex1, compared to mES cells (Figure 3), suggesting that they had undergone at least partial reprogramming to the mES cell state.

A number of previous iPS studies involved the generation of only partially reprogrammed iPS cells (pre-iPS cells) through key intermediate steps [34], and they did not express pluripotency markers, such as Oct4 and Nanog [35,36,37]. A number of typical markers of pluripotency, i.e., alkaline phosphatase or SSEA1 expression, have been shown to mark partially and faithfully reprogrammed cells, while Nanog expression was found to be mandatory in the inauguration of pluripotency in vitro and in vivo rather than maintenance of the pluripotent state per se [38].

**Figure 4:**
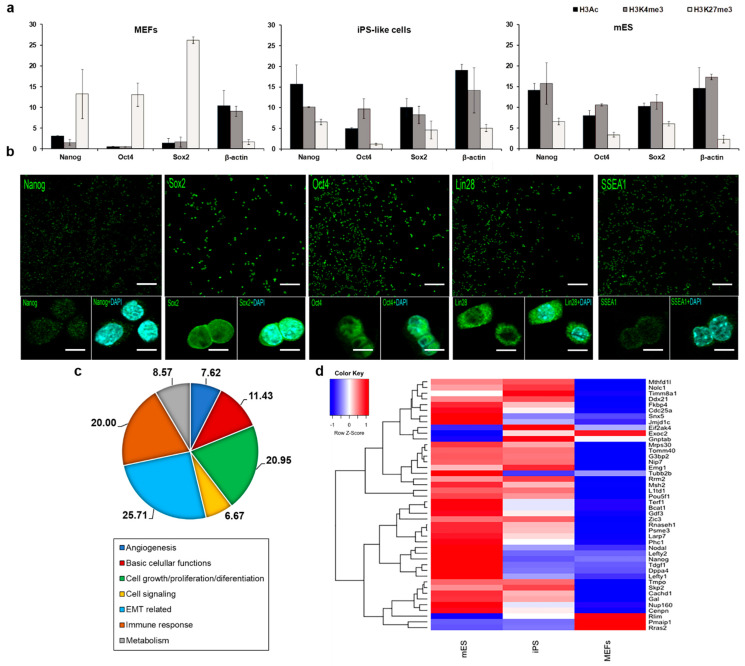
Expression of pluripotency in iPS-like cells. (**a**) Analysis of histone modification levels at β-actin (housekeeping gene), Nanog, Oct4, and Sox2 in MEF, iPS-like cells, and mES. The qPCR data are presented as a percentage of input DNA. Data were quantitated in duplicate for at least three separate experiments (mean ± SEM). (**b**) Expression of pluripotency (Nanog, Sox2, and Oct4) and embryonic (Lin28, SSEA1) related genes in iPS-like cells were revealed by immunostaining. Scale bars = 100 μm (**upper panel**) and 20 μm (**lower panel**). (**c**) GO analysis describing the main functions of the iPS-like cells. (**d**) Analysis of pluripotency-related genes contained in the microarrays showed that iPS-like cells were more similar to mES cells than MEFs regarding their pluripotent state.

As epigenetic changes have been demonstrated to be critical for reprogramming chromatin immunoprecipitation (ChIP), real-time RT-PCR was used to comparatively analyze gene-specific active (H3K4me3 and H3ac) and repressive (H3K27me3) histone modification levels at the pluripotency marker genes Nanog, Oct4, and Sox2 in mES, iPS-like cell lines, and MEF (Figure 4a, Table S2). In mES and in our reprogrammed cells, whereas H3K27me3 levels were low, H3K4me3 and H3Kac levels were highly abundant at the promoter regions of the pluripotency genes Nanog, Oct4, and Sox2. In contrast, in MEF cells, the promoter of the three pluripotency marker genes displayed a strong enrichment for the repressive H3K27me3 modification. MEFs over-expressing E1a-12S showed epigenetic characteristics that were more similar to those of ESCs than to the parental cell type. Interestingly, a similar epigenetic transformation had been previously reported [18,19], supporting the involvement of this adenoviral gene in the reprogramming process.

The pluripotent characteristics of IPS-like cells were also compared to mES cells by immunocytochemistry. Both cell lines showed positive staining for the pluripotency markers studied (Figure 4b), consistent with previous studies [39]. In previous reports, E1a has been shown to transcriptionally activate the expression of two of the Yamanaka factors, Oct4 and c-Myc [12,13,14,15]. The activation of Oct-4 in differentiated cells by E1a occurs via an interaction with TATA box factors [14]. Additionally, the E1a binding to the retinoblastoma (Rb), p300, and p400 proteins has been involved in c-Myc activation [12,13], leading to progression through to the S phase and the loss of growth control [13,17]. In this sense, a recent study has shown the generation of iPS mediated by the reprogramming cocktail composed of Oct4, Klf4, and E1a displaying similar results to ours regarding the endogenous expression of pluripotency molecular markers [25]. Finally, the epigenetic reprogramming induced by E1a has also been shown to be due to its binding to p300 [18,19]. In addition to these roles, the strong Sox2 and Klf4 expression observed in E1A-induced iPS-like cells indicates that this adenoviral protein can also activate, directly or indirectly, the expression of these factors. Those suggested potential mechanisms by which E1a-12S could reprogram somatic cells.

Moreover, genome-wide profiling by microarray analysis showed a closer gene regulation between mES and iPS-like cells than between MEFs. At least 1860 genes were closer to mES than to MEFs when compared among them (Figure 4c and Figure S4 and Table S4). Hierarchical clustering of microarray data identifies two major branches, one containing iPS-like and mES expression profiles and another apart containing only those of MEF (Figure 4d and Figure S4). Particularly, iPS-like cells share a higher percentage of pluripotent genes with mES cells than with MEFs [40]. KEGG pathways (Figure S4c) from upregulated genes showed that the same pathways were activated when comparing mES and iPS-like cells vs. MEFs. On the other hand, a comparison between iPS-like cells and MEFs vs. mES cells illustrated the activation of different pathways, demonstrating a higher similarity between iPS-like and mES cells than MEFs. Hence, the genetic programs triggered by iPS-like and mES cells are more alike than those induced by MEF. Further validation by RT-qPCR of some representative genes (Il6, Lox, Loxl1, Thbs1, Vcam1) confirms this close relationship (Figure S4d).

### 3.3. iPS-like Cells Can Be Differentiated into Three Germ Layers In Vitro

The analysis of the expression of differentiation-related genes by RT-qPCR (Table S3) in E1a-iPS derived EBs showed an upregulation in the expression of markers of all three germ layers, confirming the pluripotent nature of the reprogrammed cells (Figure S3b). Compared to control samples (mES-derived EBs), endoderm (Gata4 and Gata6) and mesoderm (Flk1 and T) related genes were slightly upregulated (<0.25 fold) in EBs generated by iPS-like cells, while ectoderm markers (Nestin, Pax6, and Sox1) were significantly upregulated. The expression of Nestin, Sox1, and Pax6 markers of neural and epidermal lineages was significantly higher than that observed in mES-derived EBs, most notably in the case of Pax6, which was overexpressed by about 16-fold.

**Figure 5:**
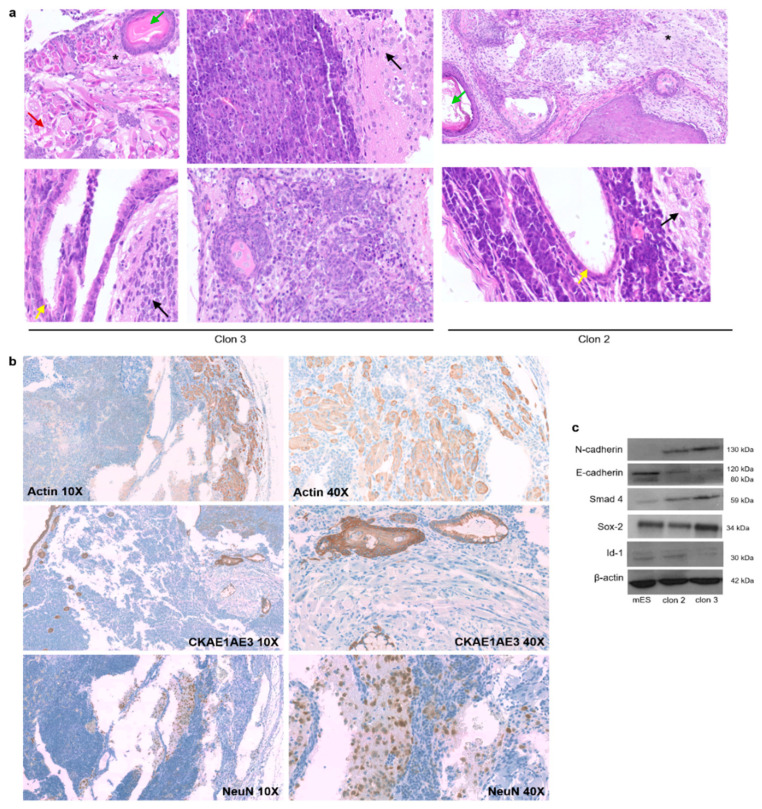
Differentiation potential of iPS-like cells: (**a**) Bright-field images of hematoxylin and eosin staining of histological sections obtained from teratomas generated by iPS-like cells (40×). Arrows indicate keratinized epithelium (green), muscle (red), ciliated epithelium (yellow), nervous tissue (black), and neurons (*). Black asterisk indicates the presence of neurons. (**b**) IHC staining of histological sections obtained from teratomas generated by iPS-like cells. Images at 10× and 40× for Actin, NeuN, Vimentin, and Cytokeratin AE1/AE3 (pankeratin). Pictures show keratinized and ciliated epithelium (CKAE1/AE3), muscle (actin), and nervous tissue and neurons (NeuN) (**c**) Expression of EpiSCs-related markers (Smad4 and Id1), epithelial marker genes (E-cadherin), and mesenchymal marker genes (N-cadherin).

Finally, the in vivo development potential of iPS-like cells was tested. First, we studied teratoma formation in nude mice, and all the subcutaneous injections yielded teratomas. Haematoxylin and eosin staining of paraffin-embedded sections revealed that these cells were able to differentiate into cell types generated from the three germ layers (Figure 5a).

Then, we tried to determine whether iPS-like cells could generate full-term chimeric mice. Reprogrammed cells were injected into C57BL/6JOlaHsd blastocysts, and eight cell embryos were transferred to the uterus of pseudo-pregnant females. Prior to the chimera analysis, the karyotype of the cells was studied (Figure S5). We obtained 81 pups, and all of them showed uniformly black coat color, indicating the absence of chimerism from iPS-like cells. These data indicate that the iPS-like cells were not able to generate chimaeras, though the differentiation potential of our reprogrammed cells was clearly demonstrated as they can be differentiated in cells from the three germ layers as the in vitro differentiation assays and the generation of teratomas revealed. With this scenario, our iPS-like cells clearly resemble EpiSCs, as it is shown in Figure 5b. EpiSCs are in vitro and in vivo pluripotent cells, though unable to contribute to chimerism, exerting a late developmental phase of pluripotency [41]. This characteristic agrees with our results, as our cells showed the same behavior and expressed the key EpiSCs markers, Id1 and Smad4, and the opposite expression of the epithelial marker gene E-cadherin and the mesenchymal marker gene N-cadherin [42].

## 4. Discussion

Reprogramming somatic cells into iPS cells can be achieved by transient forced expression of a combination of the exogenous transcription factors Oct4, Sox2, Klf4, and c-Myc (OSKM). In the past decade, novel approaches for iPS generation have been developed in an effort to elucidate the molecular mechanism of the process and to in-crease the efficiency of reprogramming [43,44]. For instance, small molecules can target signaling pathways involved in the regulation of gene expression levels of pluripotent transcription factors, inducing reprogramming of somatic cells or leading to sustained pluripotency [45,46]. In this context, E1A, the first protein coding gene ex-pressed after adenovirus infection, interacts with many cellular proteins, including tumor suppressor proteins and chromatin remodeling proteins, to organize the expression of all other viral transcripts and to initiate the reprogramming of the infected cell to allow viral replication and growth [47,48]. Different studies have pointed out the direct and indirect association of E1a and the Yamanaka factors Oct4, c-Myc and Sox-2 in the regulation of the cell cycle and the repression of cellular differentiation. 

We observed that forced expression of E1a-12S in MEFs induced partial reprogramming, resulting in, what we termed, iPS-like cells with an ESC like morphology, alkaline phosphatase staining, robust Oct4, Sox2 and Klf4 expression, and an overall gene expression profile that clustered closer to ESCs than MEFs. Moreover, our iPS-like cells were capable of forming colonies and generating embryoid bodies with an upregulated expression of markers of all three germ layers, pointing to a clear modification of MEF cells to a pluripotent-like cell lineage. Also, when induced to form teratomas, iPS-like cells could give rise to cell types derived from the three embryonic germ layers. In addition, ChIP assays, immunocytochemistry and GEO analyses highlighted that the genetic programs triggered by iPS-like cells and mES cells are more alike than those induced by MEFs. Interestingly, a similar epigenetic transformation had been previously reported [18,19]. However, RT-qPCR experiments and microarray analysis presented low levels of Nanog and Rex1 expression, and together with the absence of contribution to chimaeras, did not point to a strong reprogramming of cells by E1a retro-viral gene. These results highlight the closer resemblance of our iPS-like cells to EpiSCs, where similar patterns are observed. 

Furthermore, even though two small molecule inhibitors were employed to maintain pluripotency, the efficiency of the process was found to be lower compared to other methods [49,50]. The replacement of exogenous Sox2 by E1a has been recently shown to intervene in the reprogramming process together with Klf4 and Oct4, even though E1a alone or with Oct4 did not result in the pluripotent state [25]. Of note, the mentioned study was performed with an E1a-CR1 deleted mutant, in which direct binding to p300 [51], as well as indirect interactions with factors such as Sox-2 [16,52,53], are lacked. As we hypothesize that many of the E1a effects are due to the binding to pRb, p105 or p300, the differences between both studies might be due to the deletion on such an important region in E1a. Together with these studies, our work highlights the involvement of this adenoviral gene in the reprogramming process 

In summary, our study has shown that over-expression of the adenoviral protein E1a-12S in somatic cells was sufficient to induce partial reprogramming of the basal status of MEF cells, obtaining a cell state that resembles that of EpiSCs. Although E1a acts as a transcriptional regulator, controlling the expression of viral and cellular genes, the resulting partial reprogramming may be related to the use of the smaller isoform of the E1a gene (12S). The adenovirus E1a gene can be spliced into two different isoforms: 12S and 13S. The 13S isoform contains an additional protein domain called the CR3 domain, which allows the interaction with different cellular proteins, such as the tumor suppressor protein p53, whereas the 12S isoform does not. Moreover, the E1 locus presents another region called E1b. The E1b gene of adenovirus codes for a protein called early region 1B, which is important for viral replication and modulation of the host cell environment during infection. E1b has been shown to interact with several cellular proteins, including p53 or the MRN (Mre11-Rad50-Nbs1) complex, which plays a critical role in repairing double-stranded DNA breaks and maintaining telomere length [54]. Previous studies have shown that p53 plays a critical role in the reprogramming of iPS cells. During the reprogramming process, p53 can be activated by different stimuli, such as DNA damage or oxidative stress, which may induce cell cycle arrest or apoptosis to prevent the formation of iPS cells with DNA damage or chromosomal abnormalities. However, the excessive activation of p53 can also inhibit the generation of iPS cells. Therefore, the inhibition of p53 activity or the MRN complex (also, by the adenoviral CR3 or E1b proteins) may promote the generation of iPS cells with higher efficiency, as well as increase the quality of the resulting iPS cells [55,56]. Lastly, a possible explanation for the lack of chimeras would be that pRb blocking by E1a might avoid brain development on the fetuses as previously de-scribed [57]. 

Our findings raise important implications for the involvement of viral genes in cancer forming cells. The link between viruses and cancer has been known for decades, such as the involvement of human papiloma virus (HPV) in cervical cancer [58] or human hepatitis B virus (HBV) in hepatocellular carcinoma [59]. The E1a gene has been suspected as an oncogene since the early 80s and its neoplastic effects are most clearly demonstrated when coexpressed together with another oncogene such as Bcl2, or its counterpart E1b19k [60,61]. The fact that the molecular mechanisms underlying the generation of iPS cells are remarkably similar to those that are deregulated in cancer, to the extent that aberrant reprogramming can be directly linked to tumorigenesis [62,63,64], points to the possibility that at least part of the oncogenic activity of certain proteins may be directly due to their ability to activate the pluripotent gene regulatory network and in this way generating a pre-neoplastic lesion. Our results show that forced expression of E1a in differentiated cells is sufficient to induce partial reprogramming when these cells are placed under the right culture conditions. This finding provides a direct mechanistic link between viral reprogramming and the generation of cancer stem cells. 

## Data Availability

Microarray raw data tables have been deposited in the Gene Expression Omnibus (submitter G.M.-B.).

## Supplementary Materials

The following supporting information can be downloaded at: https://www.mdpi.com/article/10.3390/cells12091338/s1, Table S1: E1a nested PCR primers’ sequences; Table S2: TaqMan^®^ Gene Expression Assays used in the detection of pluripotency gene expression in mES, iPS-like cells, and MEF, and in the validation of the microarray analyses; Table S3: qPCR primers’ sequences used in the GeneChIP assay and in the detection of differentiation markers expression in embryoid bodies (EBs); Table S4: qPCR primers’ sequences used in the GeneChIP assay and in the detection of differentiation markers expression in embryoid bodies (EBs); Table S4: Genes modified (at least 2-fold) in MEF, iPS-like, and mES cells. Figure S1: Transformation process of MEF during reprogramming; Figure S2: Characterization of iPS-like cells; Figure S3: Characterization of embryoid bodies; Figure S4: Microarray analysis of the expression of pluripotency in iPS-like cells; Figure S5: Karyotype analysis of various iPS-like cells. Supplemental Experimental Procedures: Retrovirus production, Cell proliferation assay, Cell cycle analysis, In vitro differentiation assays, Western blot.
