# Supplementary material for: The E1a Adenoviral Gene Upregulates the Yamanaka Factors to Induce Partial Cellular Reprogramming
Source: Cells. 2023 May 7;12(9):1338. doi: 10.3390/cells12091338 (PMC10177049; doi:10.3390/cells12091338)
Supplement: Supplementary file 1 [file cells-12-01338-s001.zip › cells-2215530-supplementary.pdf]

Supplemental Material

The E1a Adenoviral Gene Upregulates the Yamanaka Factors to Induce Partial Cellular Reprogramming

SUPPLEMENTARY TABLES

Table S1. E1a nested PCR primers' sequences. Related to Figure 2.

Gene	Primer F	Primer R
E1a (1st amplification)	TTAGATTATGTGGAGCACCCC	ACAGCTATCCGTACTACTATTGCAT
E1a (2nd amplification)	TTAGATTATGTGGAGCACCCC	TCCTCATATAGCAAAGCGAAC

Table S2. TaqMan® Gene Expression Assays used in the detection of pluripotency gene expression in mES, iPS-like cells and MEF, and in the validation of the microarray analyses. 18S was used as housekeeping gene in both approaches. Related to Figure 3.

	Gene	ID Assay
PLURIPOTENCY	c-Myc	Mm00487804_m1
	Klf4	Mm00516104_m1
	Nanog	Mm02384862_g1
	Oct4	Mm03053917_g1
	Rex1	Mm00617735_m1
	Sox2	Mm03053810_s1
	18S	Mm01974474_gH
MICROARRAY VALIDATION	Ahsg	Mm00442674_m1
	Fgf4	Mm00438916_g1
	Il6	Mm01210733_m1
	Lox	Mm00495386_m1
	Loxl1	Mm01145738_m1
	Tcfcp2l1	Mm00470119_m1
	Thbs1	Mm01335418_m1
	Vcam1	Mm00449197_m1

Table S3. qPCR primers' sequences used in the GeneChIP assay and in the detection of differentiation markers expression in embryoid bodies (EBs). β-actin was used as housekeeping gene. Related to Figure 3 and supplementary Figure 2.

	Gene	Primer F	Primer R
GeneChIP ASSAY	β-actin	GATGCTGACCCTCATCCACT	ACTGCCCCATTCAATGTCTC
	Nanog	GCTTGAACCAGCCAGTTCTC	GGGCGACGTAATTTTGGTAA
	Oct4	CTCCTCCACCCACCCAGGGG	GGGCCTGGTGGAAAGACGGC
	Sox2	GGTGAGAAGAGGGGGTGAGT	GTGGTGTGCCATTGTTTCTG
DIFFERENTIATION MARKERS EBs	β-actin	GGCCCAGAGCAAGAGAGGTAT	ACGCACGATTTCCCTCTCAGC
	Flk1	AGAACATTTGTCCGAGTTCACA	CGGACTTGACTGCCCACT
	Gata4	GAGGCTCAGCCGCAGTTGCAG	CGGCTAAAGAAGCCTAGTCCTTGCTT
	Gata6	GACTCCTACTTCCTCTTCTTCTAATTCAGA	ACCTGAATACTTGAGGTCACTGTTCTC
	Nestin	CTGCAGGCCACTGAAAAGTT	TCTGACTCTGTAGACCCTGCTTC
	Pax6	CCATCCTTTGCTTGGGAAATCC	GCTTCATCCGAGTCTTCTCC
	Sox1	GCGAGATGATCAGCATGTACC	TAGTGCTGTGGCAGCGAGT
	T	CGACCACAAAGATGTAATGGAG	CAGCACCAGGAACAAGC

Table S4. Genes modified (at least 2-fold) in MEF, iPS-like and mES cells. Related to Figure 4.

Gene	MEF	IPS-like cells	mES
EMT related (n=28)			
**Acta1**	11.85	6.32	5.81
**Acta2**	16.33	9.67	6.29
**Actg2**	14.97	7.51	5.96
**Col12a1**	11.65	6.46	5.85
**Col1a1**	14.91	5.86	6.58
**Col1a2**	15.99	7.02	7.31
**Col3a1**	13.46	5.77	5.73
**Col6a1**	13.03	6.08	5.70
**Col8a1**	11.55	5.81	5.81
**Ecm1**	13.93	7.51	7.19
**Efemp1**	11.79	6.66	5.98
**Emcn**	11.43	5.81	5.83
**Fgf7**	12.09	6.78	6.23
**Itgal1**	15.67	5.79	5.90
**Lox**	12.49	5.84	5.76
**Loxl1**	13.37	5.84	6.70
**Ltbp2**	15.79	6.04	6.26
**Mmp3**	11.38	5.89	5.93
**Nexn**	11.35	5.94	5.94
**Ogn**	11.74	5.77	5.68
**Postn**	11.46	6.86	5.83
**Prrx2**	12.56	5.86	6.53
**Selp**	12.79	6.16	6.05
**Serpine1**	15.69	5.86	7.82
**Svep1**	12.77	6.90	6.54
**Thbs1**	15.69	7.76	5.89
**Thbs2**	12.97	6.40	5.89
**Timp3**	15.74	6.60	5.76
Cell growth/proliferation/differentiation (n=21)			

**Ahsg**	5.90	10.69	11.20
**Dbn1**	12.66	6.68	6.24
**Dcn**	13.54	5.81	5.71
**Egr1**	13.40	6.27	5.90
**Ereg**	11.27	5.70	5.51
**Fgf4**	5.76	11.53	14.29
**Fibin**	10.69	5.83	5.70
**Flt1**	12.22	6.01	6.15
**Ghr**	12.42	7.48	6.24
**Glipr1**	11.45	6.07	5.79
**Grem1**	13.40	6.90	5.68
**Ifi202b**	12.35	6.43	5.68
**Ifi204**	11.74	6.78	5.79
**Ifit3**	11.55	6.13	5.62
**Inhba**	14.19	5.79	5.64
**Mgp**	14.13	5.81	5.88
**Rspo2**	13.01	5.73	5.95
**Tcfcp2l1**	5.79	10.11	13.50
**Twist2**	11.26	5.67	5.77
**Wisp1**	12.89	5.89	5.88
**Wisp2**	11.16	5.81	5.98

SUPPLEMENTARY FIGURES

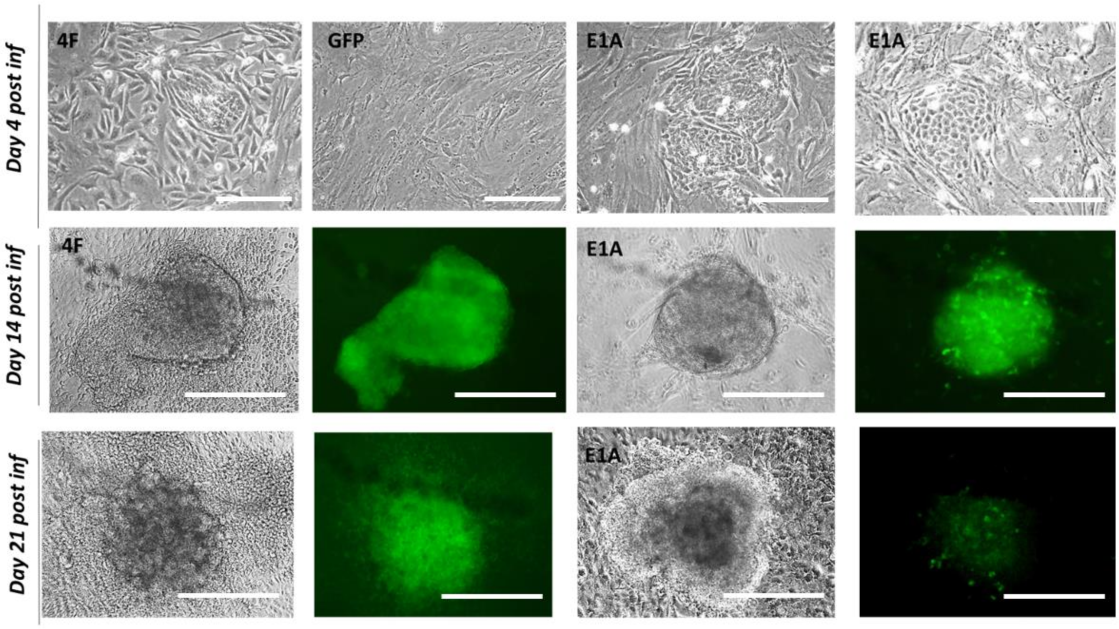

Figure S1. Transformation process of MEF during reprogramming. MEFs were initially infected either with a retrovirus carrying E1a-12S, GFP or the 4 Yamanaka´s factors (4F). On the top row, changes produced (except for GFP infected cells) at 4 days post-infection are shown, when cells were cultured with reprogramming medium. The intermediate and bottom rows show changes on the E1a-12S and 4F cells at different time-points of the process (14 and 21 days after infection). As MEF Oct4-GFP were used, fluorescence was observed along the process.

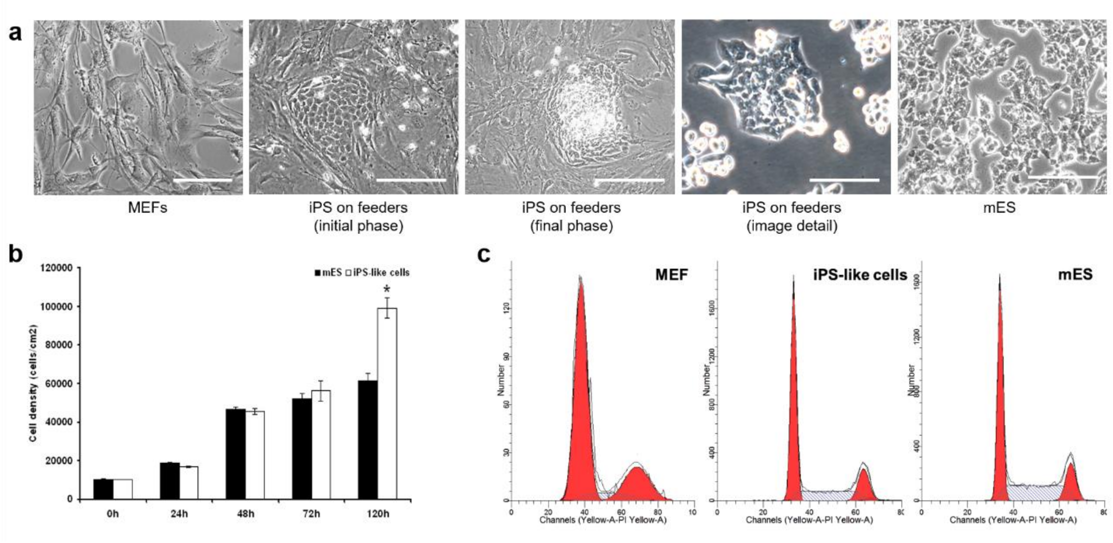

Figure S2. Characterization of iPS-like cells. (a) Phase-contrast images of MEF Oct4-GFP (left) before E1a retroviral reprogramming, iPS-like cells (middle) after at different times in their conditioned medium, and mES cells (right). E1a-transduced cells can generate colonies on gelatinised culture plates changing their morphology and resembling more to mES cells. Scale bars 200μm. (b) Cell density (cells/cm^2) was monitored for 5 days (120 h) to test the cell proliferation differences between mES and iPS-like cells. Values are mean ± SEM (n = 3). (c) Cell cycle analysis by flow cytometry in MEF (left), iPS-like cells (middle) and mES cells (right) showed the close proximity of iPS-like cells to mES.

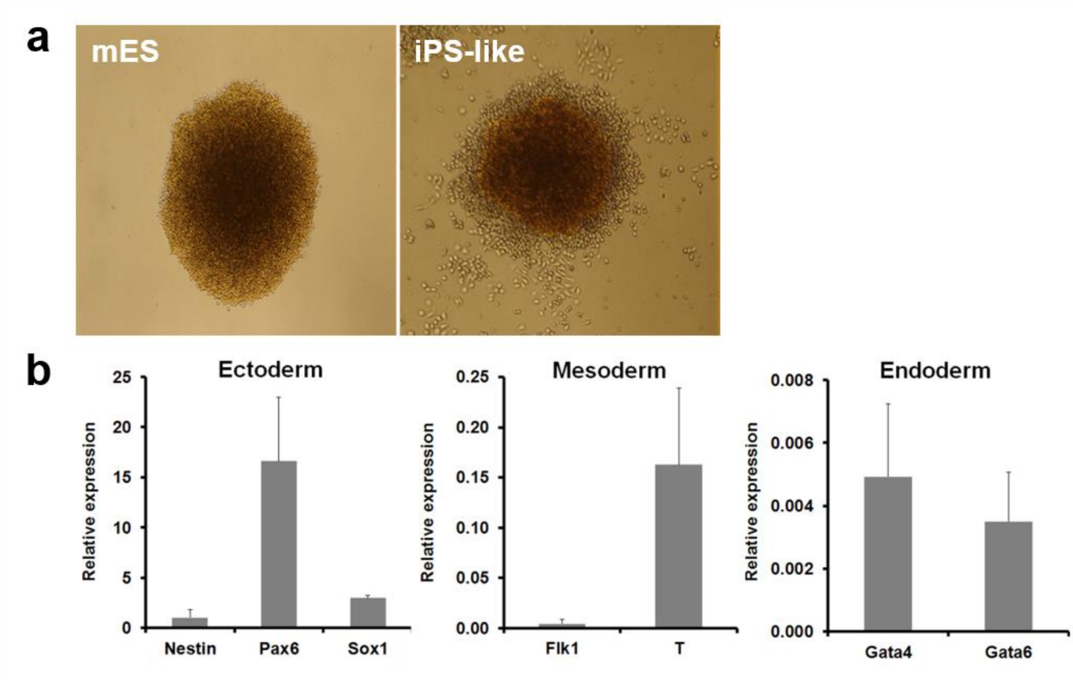

Figure S3. Characterization of embryoid bodies. (a) Bright-field images of adhered embryoid bodies of mES (left) and iPS-like cells (right). Scale bars 100µm. (b) qRT-PCR of gene markers of the three primary germ layers in EBs in differentiation. Expression values were normalized to the housekeeping β-actin gene and related to control sample (mES) expression (RQ = 1). Error bars represent mean ± SEM (n = 3).

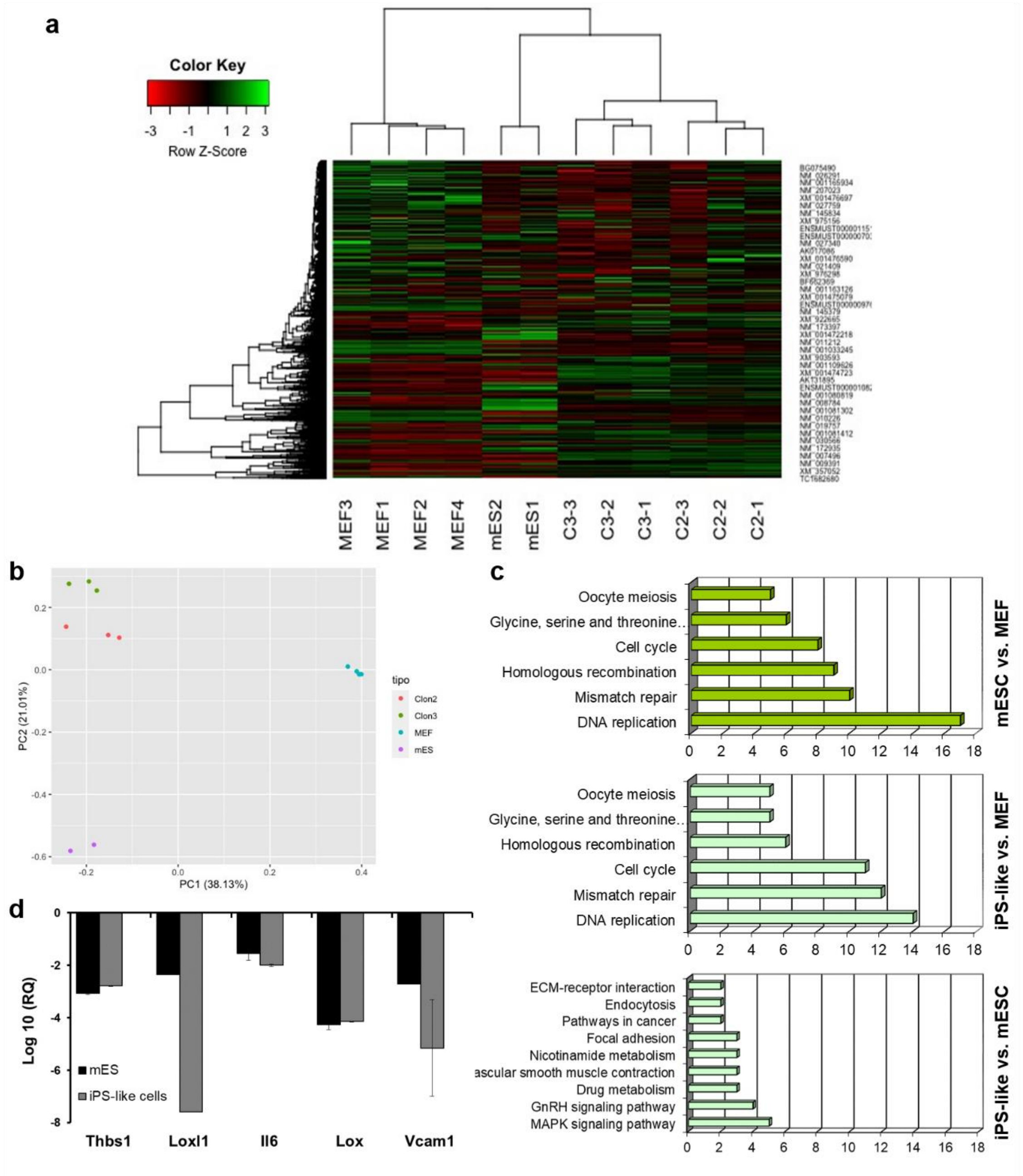

Figure S4. Microarray analysis of the expression of pluripotency in iPS-like cells. (a) Microarray analyses showed a close global gene expression profile between iPS-like cells and mES, which were very different from that exerted by MEF. C2-1 to C-3 represent different iPS-like clones. Cluster analysis classified the samples into groups based on the expression levels in each sample. The dendrogram shows significantly different gene expression levels among samples. Red indicates high expression of the namely gene, and green indicates relatively low expression. (b) PCA plot shows clusters of samples based on their similarity. (c) KEGG pathways from upregulated genes compared between mES cells, iPS-like cells and MEFs. (d) Validation by RT-qPCR of some representative genes (Il6, Lox, Loxl1, Thbs1, Vcam1). Expression values were normalized to the housekeeping

18S gene and expressed related to the control sample (MEF) expression (log(RQ) = 0, where RQ = relative quantification). Error bars represent mean ± SEM (n = 3).

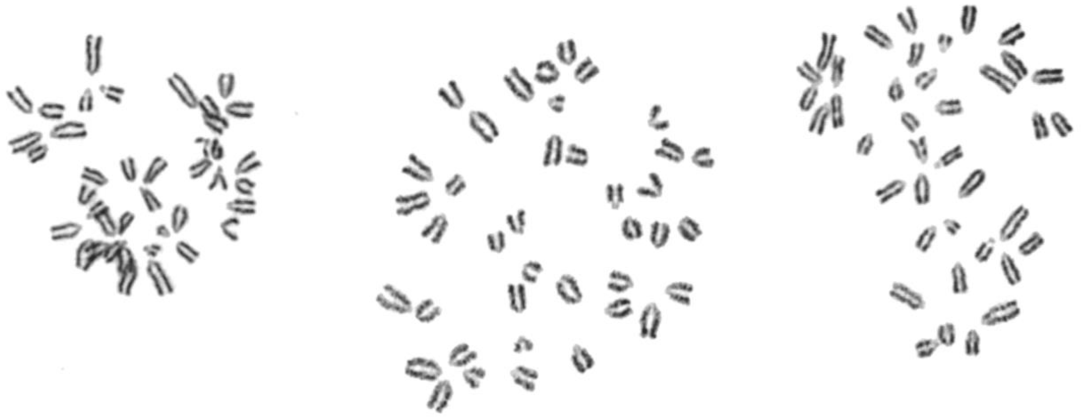

Figure S5. Karyotype analysis of various iPS-like cells. 40 mitosis were analyzed and less than 10% presented the correct chromosomal number. The rest of iPS-like cells were aneuploid, presenting only 39 chromosomes.

SUPPLEMENTAL EXPERIMENTAL PROCEDURES

Retrovirus production

Packaging 293T cells were plated the day before transduction on 10 cm dishes to be 70-80% confluent upon transfection. On the next day, 1 ml of serum-free DMEM containing 20 µg of the expression plasmid pLPC-E1a-12S and 10 µg of helper plasmid was prepared for each plate. As transfection reagent, linear 25 kDa polyethilenimine (PEI) was used. The serum free DMEM mixture was supplemented with 75 µl of PEI (1 mg/ml). This mix was incubated for 30 min at room temperature before addition onto the cells drop by drop. Cells were incubated and 48 h after transfection, supernatant was collected and filtered through a 0.45 µm pore membrane (Nalgene). The expression plasmid pLPC-E1a-12S was kindly donated by Dr. Ramón y Cajal (Hospital Vall d'Hebron, Barcelona, Spain). The pLPC-E1a-12S plasmid was first described in a previous work by Samuelson et al (Proc Natl Acad Sci U S A. 1997; 94(22):12094-9) and the E1a-12S gene was described in the reference of Stephens et al (The Embo Journal 6:2027-2036, 1987). More information could be obtained at the Addgene site (https://www.addgene.org/18740/sequences/).

Cell proliferation assay

mES and iPS-like cells were seeded on gelatin coated 96-well plates at 5000 cells/well in their appropriate supplemented media. Cell proliferation was determined by the alamar blue assay (O'Brien et al., 2000), which monitors the fluorescence that develops alamar blue (resazurin), a membrane-permeable, non-fluorescent, blue phenoxazin-3-one dye, is taken up by cells and reduced by cellular oxidoreductases to pink, highly fluorescent resorufin by metabolically active cells. Cell density was monitored at 0, 24, 48, 72 and 120 h after seeding.

Briefly, aliquots of 10 µl of stock alamar blue solution (Invitrogen) were added to each well containing 100 µl of medium and incubated with the cells for 4 h at 37ºC and 5% CO_2. Following incubation, the fluorescence was read on a plate reader at 530/590 nm excitation/emission (Biotek Synergy HT) and results were displayed by the Gen5 10.9 Data Analysis Software (Biotek).

Cell cycle analysis

Cell cycle analysis using flow cytometry was carried out to determine whether E1a transduction induced growth arrested mice cells to enter the cell cycle and begin cellular

DNA replication, and to test the differences in cell cycle between MEF, mES and iPS-like cells. Cells were washed twice with PBS, collected by trypsinization and centrifuged. The pellets containing 10^6 cells were resuspended in 200 µl PBS and 1 ml cold 70% ethanol and incubated at 4°C for 24 h. After centrifugation, the cells were washed twice and resuspended in 1 ml PBS, containing RNase (100 µg/ml; Sigma) and propidium iodide (50 µg/ml; Sigma), and incubated in the dark for 30 min. Cells were analyzed using FACS Array (Becton Dickinson) and their distribution in the different phases of the cell cycle was analyzed with MODFIT LT 3.0 (Verity Software).

***In vitro* differentiation assays**

In order to evaluate pluripotency of iPS-like cells *in vitro*, the formation of embryoid bodies in differentiation and the evaluation of gene markers of the three primary germ layers by qRT-PCR were performed.

The methodology to generate embryoid bodies in differentiation is the same as described above, but the medium was not fully conditioned because LIF and MEK and GSK3β inhibitors were not added to monitor their differentiation patterns. Thus, after 7-10 days embryoid bodies were adhered to the cell culture dish, embryoid bodies were harvested and RNA was extracted to carry out *in vitro* differentiation qRT-PCR studies as described above. Primer sequences (Sigma) are listed in Supplementary Table 4.

Western-blot

Protein extracts from all the samples (50 µg of protein) were diluted 6:1 in loading buffer and heated ay 37ºC for 30 min prior to SDS 12%-PAGE electrophoresis. Protein were electrotransferred to PVDF membrane previously activated with methanol 1 hour at 100 mA in the Tris-glicine buffer supplemented with 0.05% lithium dodecyl sulfate. PVDF membranes were blocked with 5% non-fat dry milk in Tris-buffered saline (TBS: 137 mM NaCl, 2.4 mM KCI, 25 mM Tris, pH 8.0) for 1 hour, incubated with the corresponding primary antibody for 4 hours with a 1:500 dilution of the following antibodies: anti-Ecadherin (clon 4A2C7 from Zymed), N-cadherin (3B9, Thermo Scientific 33-3900), anti-Smad 4 (B-8, SC-7966, Santa Cruz Biotechnology), anti-Id1 (C-20, SC-488, Santa Cruz Biotechnology), anti-FGF-2 (147, GSC-79-6, Santa Cruz Biotechnology), anti-SSEA1 (1:1000; SSEA1 Monoclonal Antibody (eBioMC-480 (MC-480), eBioscience™), Nanog (1:1000; eBiosciences), α-Tubulin (1:2000, Cell Signalling), Sox2 (1:100, R&D), Oct4 (Novus Bio. NB100-2379SS).and/or 1:1000

dilution of anti-actin (A2228, Sigma Aldrich), wash 3 times with TBS-1% Tween 20 for 10 min, incubates with horse radish peroxidase (HPR)-linked secondary anti-mouse (diluted 1:2000), anti-rabbit (diluted 1:1000), anti-rat (diluted 1:1000) and anti-goat (diluted 1:5000) Abs from Santa Cruz Biotechnology (Santa Cruz Biotechnology, Inc., USA). Proteins were visualized using the enhanced chemiluminiscence (ECL) western blot detention system (Thermo Scientific, IL, USA).
